# Age-related changes in neural functional connectivity and its behavioral relevance

**DOI:** 10.1186/1471-2202-13-16

**Published:** 2012-02-14

**Authors:** Winfried Schlee, Vera Leirer, Iris-Tatjana Kolassa, Nathan Weisz, Thomas Elbert

**Affiliations:** 1 Clinical & Biological Psychology, Institute of Psychology and Education, University of Ulm, Albert-Einstein-Allee 47, 89081 Ulm, Germany; 2 Clinical Psychology & Neuropsychology, University of Konstanz, Universitätsstr. 10, 78457 Konstanz, Germany; 3 Zukunftskolleg, University of Konstanz, Box × 916, 78457 Konstanz, Germany

## Abstract

**Background:**

Resting-state recordings are characterized by widely distributed networks of coherent brain activations. Disturbances of the default network - a set of regions that are deactivated by cognitive tasks and activated during passive states - have been detected in age-related disorders such as Alzheimer's or Parkinson's disease but alterations in the course of healthy aging still need to be explored.

**Results:**

Using magnetoencephalography (MEG), the present study investigated how age-related functional resting-state brain connectivity links to cognitive performance in healthy aging in fifty-three participants ranging in age from 18 to 89 years. A beamforming technique was used to reconstruct the brain activity in source space and the interregional coupling was investigated using partial directed coherence (PDC). We found significant age-related alterations of functional resting-state connectivity. These are mainly characterized by reduced information input into the posterior cingulum/precuneus region together with an enhanced information flow to the medial temporal lobe. Furthermore, higher inflow in the medial temporal lobe subsystem was associated with weaker cognitive performance whereas stronger inflow in the posterior cluster was related to better cognitive performance.

**Conclusion:**

This is the first study to show age-related alterations in subsystems of the resting state network that are furthermore associated with cognitive performance.

## Background

It is becoming increasingly acknowledged that effective information processing crucially depends on the integrity of communication between distributed cortical and subcortical regions. Deviating network patterns have been identified in mental disorders (e.g. [1,2]) and also dementia [3-5]. However, a growing amount of structural and functional evidence implies that even through normal aging dramatic changes in brain networks occur. Total brain volume declines with age [6] with evidence for changes in cingulate sulci, hippocampus, insula, caudate, cerebellum and the entorhinal cortices [7,8]. Atrophy has been documented for gray and white matter [9-12] as well as loss of synaptic connections [13]. Amyloid deposition can be observed even in non-demented elderly [14-16] in association with an aberrant default network functional activity as measured by means of fMRI [17]. However, hemodynamic measures are very slow, in the range of seconds, and do not directly capture large parts of relevant cortical activities which however unfold in the range of milliseconds and are frequently expressed in oscillations [18]. MEG is becoming an increasingly important non-invasive tool to investigate the dynamics of brain networks, since it has the temporal resolution needed to cover major bands of oscillatory brain activity as well as an increasingly improving spatial resolution due to advances in electromagnetic sourceimaging. We employed magnetoencephalography (MEG) to analyze age-related changes in connectivity up to 100 Hz. Identified network changes were tested for behavioral significance by correlating them with neuropsychological test performance.

A beamforming technique was used to reconstruct the magnetic brain activity in the source space and the strength of coupling between different areas was investigated using partial directed coherence (PDC). PDC is an approach to measure coupling between multivariate time series that is related to the concept of Granger causality [19] and captures the direction of information flow in the frequency domain [20,21].

As an interconnected system, a network consists of nodes (here: voxels) and connections between them (here: coherence). The importance of a node within the network varies among other things with the number of connections it entertains with other nodes. Accordingly, a node with a larger number of links (called hub) receives information from many other nodes and/or influences activity in many other nodes. In directed networks, the information on the directionality of the information flow is retained. The "inflow" to a voxel indicates that the activity of this voxel is driven by other voxels. Similarly, the "outflow" describes the influence of this voxel onto oscillatory activity in other voxels.

Here we investigated age-related alterations in the inflow and outflow characteristics of these functional neural networks and their association with cognitive performance.

## Methods

### Participants

Fifty-three right-handed and healthy subjects (23 males and 30 females) ranging in age from 18 to 89 years (*m *= 53.06 years, *sd *= 20.07) participated in this study. Their mean education was 15 years (ranging from 10 to 22 years). Subjects were recruited by notifications posted in different locations in the Konstanz area (e.g. residential homes for the elderly, senior citizen centers, sports clubs, the campus of the University of Konstanz) and by advertisement in the local newspaper and radio station. They were paid 30€ for their participation. Exclusion criteria were a history of psychiatric disorders, a history of psychopharmacological medication, left-handedness, metal objects in the body as well as a history of severe head injuries or neurological problems (like epilepsy, strokes, brain tumors etc.). The ethics committee of the University of Konstanz approved this study.

### Procedures

Upon arrival in the laboratory, participants were familiarized with the room where MEG measurements were taken, and the study procedures and goals were clarified. All participants gave written informed consent. Afterwards subjects were screened for potential psychiatric disorders with the MINI International Neuropsychiatric Interview [22]. Subsequently, demographic data were assessed and handedness was determined using the Edinburgh Inventory [23]. Furthermore, cognitive abilities were assessed with the *CERAD-NP-plus *test battery [24] with the subtests Verbal Fluency (VF = sum score of semantic and phonemic fluency), Word List Learning (WLL), Word List Delayed Recall (WLDR), Word List Recognition (WLR), Figure Recall (FR), Trail Making Test A and B (TMT-A/B). Additionally, the Digit Symbol, the Mosaic and the Digit Span subtests of the German version of the Wechsler Adult Intelligence Scale (HAWIE-R) [25] as well as the *Benton Visual Retention Test - revised form *[26] were conducted.

After the neuropsychological assessment, MEG recordings were obtained during a 5 min resting period with eyes open. We recorded the electrooculogram (EOG) using a bipolar montage where electrodes were attached near the left and right outer cantus (horizontal EOG) and below and above the right eye (vertical EOG). For recording of the electrocardiogram (ECG), two electrodes were attached at the left lower forearm and the right collarbone. Subsequently, participants were seated in the magnetically shielded room (Vakuumschmelze Hanau) and their head shapes were digitized with a Polhemus 3 Space Fasttrack (Polhemus, Colchester, VT, USA). Five index points were determined to calculate the relative head position within the MEG sensor for source analysis. The subjects' head position relative to the pickup coils of the sensor was estimated before and after the measurement. During MEG measurement subjects were lying in a comfortable supine position and were instructed to stay awake in a resting state. They were further asked to fixate a mark on the ceiling of the magnetically shielded room and to avoid eye as well as any body movements throughout the recording to reduce artifacts. A video camera installed inside the magnetically shielded room allowed monitoring subjects' behavior and ensured compliance throughout the experiment.

MEG was recorded continuously and digitized at a rate of 678.17 Hz using a 148-channel whole head magnetometer system (MAGNES™, 2500 WH, 4D Neuroimaging, San Diego, USA). A band-pass filter of 0.1 - 200 Hz was used for data acquisition. EOG and ECG were recorded with a SynAmps amplifier (Neuroscan ™) using Ag/AgC1 electrodes. Before and after the MEG recording, the head position of the participants were measured. If the head position of the participant after the MEG recording deviated by 1 cm or more from the position prior to the scan, the scan was excluded.

### Data Analysis

For data preprocessing and most other steps of data analysis, the fieldtrip toolbox (F. C. Donders Centre for Cognitive Neuroimaging: http://www.ru.nl/fcdonders/fieldtrip) was used. First, all data were downsampled to 600 Hz and cut into epochs of 2 s duration. Prior to downsampling, the contionous data were filtered with a band-pass of 0.1 - 200 Hz. Epochs containing blinks or muscle artifacts were excluded from further analysis based on visual inspection. Second, an independent component analysis (ICA) was calculated for each individual data set to identify components reflecting cardiac activity and these components were removed for further analyses (using the logistic infomax ICA algorithm implemented in eeglab: http://sccn.ucsd.edu/eeglab/). ICA components that represent the cardiac activity were selected based on the time course of the component and their topography. Depending on the individual component structure either zero, one, or two ICA components were removed.

Afterwards, 90 2-s epochs were selected randomly from the remaining time segments and used for the following analyses. This selection was done in order to keep the number of trials constant across all subjects.

#### Source projection

In order to estimate activity in source space, we used a linearly constrained minimum variance [27] beamformer on each individual data set. The LCMV beamformer uses the covariance matrix of the signal data to construct a spatial filter that passes the signals for each time point to a predefined source while minimizing the contributions of other sources. The spatial filters were multiplied with the sensor time series, to derive the single-epoch activities. The orientations were rotated for each epoch so that the first orientation accounted for a maximum of the signal. The orientations were then averaged across epochs and applied to the signal epoch. The subsequent analysis steps were then performed on the first orientation. A voxel grid was designed to fulfill the following criteria: 1) The grid needs to cover the entire brain volume and should be as fine as possible. 2) Voxels located outside the brain volume need to be excluded from the grid. Voxels not containing any relevant brain activity would introduce noise to the multivariate auto-regressive model and lead to erroneous PDC values. 3) Voxels at the outer border of the brain volume that cover only a small percentage of the bain volume still remain part of the grid to ensure that no relevant brain activity is missed by the analysis. 4) The number of model parameters that can reliably be estimated in the autoregressive model is limited by the number of trials that are used for the analysis, the duration of the trials and the sampling rate. Increasing the number of voxels (and thus the number of model parameters) over a certain limit would result in an ill-posed autoregressive model and spurious PDC estimates. Therefore, the maximum number of voxels was limited. With respect to the criteria 1) - 4) the grid of 326 voxels and a voxel size of 2 × 2 × 2 cm was the best resolution for this type of analysis. With this voxel size, the minimum distance between the centres of two neighbouring voxels is 2 cm and the term "long-range connectivity" that we use in this manuscript thus referes to rather macroscopic brain distances of at least 2 cm.

Correlation coefficients between the beamformer weights were calculated for all possible voxel pairs. Over all participants, beamformer weigths correlated very low with a mean of 0.044 and a variance of 0.024. Furthermore, there was no significant correlation between the average correlation of beamformer weights and the participants age (r = .16, p = .25). This was an important prerequisite for the following analysis to ensure that the functional connectivities below are not a result of correlated beamformer weights.

#### Partial directed coherence

We closely followed the procedure developed and described in detail by [28]: For each subject, we computed partial-directed coherence (PDC) for the full set of voxels [21]. Partial directed coherence is a measure of effective coupling that captures the direction of the information transfer between the voxels. Thus, with a set of N voxels, we get a total of N × N PDC values for each subject that reflects for each pair of voxels the effective coupling in both directions. This approach is based on multivariate autoregressive (MVAR) modeling that integrates temporal and spatial information. Here, we model for each voxel the influence of all other voxels for a given time range. The model order defines this time range of the autoregressive process and describes how many time points - back in time - are used for modeling the current value. The optimal model parameter p was found by calculating the Schwarz Bayesian Criterion (SBC) [29] for model orders from 2 - 20. Averaged over the study sample, the minimum of the SBC function was located at p = 6 which was then taken as the model order for all subjects. Partial directed coherence is a statistical measure that is related to the concept of Granger causality [19] and is able to detect asymmetric coupling between the compared voxels for a given frequency range. In this study, for each voxel we modeled the influence this single voxel receives from all other voxels in the frequency-range from 1 to 100 Hz (increments of 1 Hz). The PDC values were calculated using functions implemented in the biosig toolbox http://www.biosig.sf.net.

There is no generally established way of calculating the statistical significance of the PDC estimators. Thus, we used a permutation approach to estimate thresholds for significant coupling between pairs of voxels (couplings of one voxel with itself were excluded from the analysis). Therefore, the following three steps were repeated 1000 times for each data set:

First, the matrix of the autoregressive coefficients was shuffled pseudo-randomly. This was done the following way: The matrix of the autoregressive coefficients is a square matrix with 326 rows and 326 columns. Therefore, we generated a vector with random numbers between 1 and 326. The columns and rows were reordered according to the random vector. Subsequently, the rows were shuffled according to the same random vector. Second, the PDC estimators were again calculated in the way that was described above. Third, we determined the 99%-percentile of the PDC estimator for each frequency and saved it. The maximum value over these 1000 permutations was used as a threshold of significance for each frequency bin. In a very recent publication, Florin and colleagues [30] systematically compared random permutation with the leave-one-out-method (LOOM) and found that random permutation with PDC values result in less false positives, however, more misses than the LOOM approach. There was a genereal effect showing that lower frequency bands engage stronger functional connectivity than the higher frequencies, which will be reported elsewhere by the same group. Due to this effect it was necessary to use different thresholds of significance for the distinct frequency bins rather than one threshold for all frequencies in order to avoid weighting the lower frequencies over the higher frequencies.

#### Hubmapping

Networks of any kind can be described by the distribution of their hubs. Within a network, the degree of a node can be calculated by the number of connections that link to other nodes in the network and nodes with a high degree are called hubs [31]. Based on the measures of partial directed coherence we constructed networks whereby the nodes in the network correspond to the brain voxels and the links correspond to the estimated functional connectivity between the brain voxels. A voxel with a high degree of connectivity therefore gives us a measure for the importance of this region within the functional brain network. In this analysis we weighted the degree of the hub by the strength of the couplings (i.e. the PDC estimator). Only significant couplings between pairs of voxels were used for the calculation of the hubs. Since Partial Directed Coherence allows an interpretation of the directionality of the coupling between two voxels we were able to differentiate between "Inflow" and "Outflow". The degree of inflow at voxel *x *is therefore a measure of how strong the activity in voxel *x *is influenced by the activity of other voxels. Likewise, the degree of outflow is a measure of how strong voxel *x *influences the activity of all other voxels. The degrees for inflow and outflow were calculated for each frequency bin separately. For statistical analysis and visualization purposes, they were mapped on a template MRI from the Montreal Neurological Institute (MNI) using a nearest-neighbor interpolation.

### Statistical analysis

#### Age-related effects

The correlation between age and inflow/outflow was calculated using a nonparametric randomization test [32,33] with the following procedure: First, a Pearson product moment correlation between inflow/outflow and age was calculated for each voxel and voxels with a p-value lower than .05 were selected. Clusters of the selected voxels were formed based on their adjacency in the frequency domain and space. The statistical significance of this cluster was tested using a randomization method that controls for type I error. A total of 1000 permutations were performed by randomization of the age values. The correlation coefficients were re-calculated for all cluster and the maximum correlation coefficient was taken. The distribution of this 1000 correlation coefficients was used to generate the t-values reported here. This statistical analysis was calculated for the inflow and the outflow degrees separately (see [28]).

#### Correlation with behavioral data

In the first step, we were interested in age-related alterations in the organization of resting state networks. Accordingly, we correlated age and inflow or outflow, respectively, and found clusters of corresponding age-related changes. Furthermore, we were interested in the correlation between the inflow and cognitive performance. Therefore, the clusters with age-related inflow changes found in the first step were defined as clusters of interest and used for further statistical analyses. To investigate the relationship between the age-related changes of the functional brain networks with the behavioral data from the neuropsychological testing we calculated Pearson's product-moment correlation coefficients between the average degree of the clusters of interest of all participants with their neuropsychological test scores. To correct for multiple comparison, we adjusted the threshold for a significant correlation to the level of *p *= 0.0045 according to the Bonferroni-method. This correlation was calculated for the average of all inflow clusters.

#### Correlation between significant inflow clusters

In order to investigate whether the clusters with age-related inflow changes found in the first step are independent of each other, the Pearson's product-moment correlation coefficient was calculated.

## Results

### Age-related differences

#### Inflow

**Figure 1:**
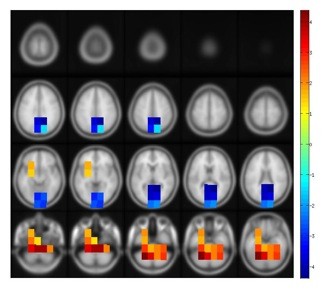
**Clusters of significant correlations between age and strength of inflow projected on a template brain**. Positive t-values (cluster 1-4 jointly illustrated) indicate that older people have more inflow in this region (mainly medial temporal lobes including hippocampus); negative t- values (cluster 5) imply less inflow for older adults. A cluster-based randomization was done in order to calculated the t-values from a distribution of 1000 randomizations.

**Figure 2:**
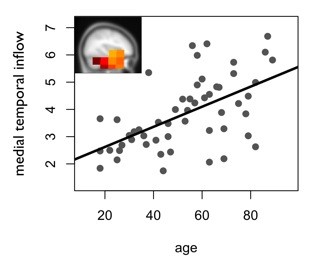
**Correlation of age and the average weighted degrees for the significant positive inflow cluster in the medial temporal lobes (r = .60, p < .001)**.

**Figure 3:**
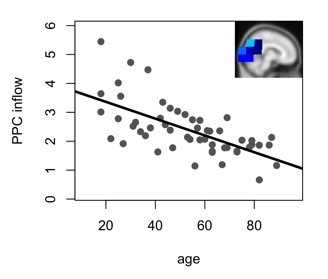
**Correlation of age and the average weighted degrees for the significant negative inflow cluster in posterior areas (r = -.65; p < .001)**.

In an analysis of the inflow of the cortical networks we found 5 clusters that showed significant age effects (Figure 1). In the clusters 1 (*p *= .01), 2 (*p *= .01), 3 (*p *= .02) and 4 (*p *= .05) inflow was significantly increased with increasing age (Figure 2). Given that the positive clusters 1-4 covered broadly the same area in the medial and inferior temporal lobes we illustrated them altogether in Figure 1 in red and yellow color. These colors symbolize positive values, implying that the inflow in this region becomes stronger with higher age. With increasing age, inflows were stronger for the 40-70 Hz (cluster 1), 8-32 Hz (cluster 2), the 85-100 Hz (cluster 3) and the 32-40 Hz (cluster 4) frequency bands. Cluster 5 was the only cluster with negative *t *values, i.e. the degree of inflow was stronger for younger compared to older individuals (p < .001). The voxels of cluster 5 overlay a large area of the posterior part of the brain. The inflow was significantly reduced for the elderly in the 1-100 Hz frequency range (Figure 1 and 3).

#### Outflow

**Figure 4:**
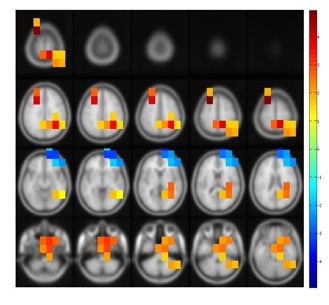
**Clusters of significant correlations between age and the strength of the outflow, projected on a template brain**. Positive t-values mean that older individuals have more outflow from this region, negative t-values imply less outflow for older individuals. A cluster-based randomization was done in order to calculated the t-values from a distribution of 1000 randomizations.

For the outflows, we found 8 clusters exhibiting significant age-related differences (Figure 4). Cluster 1 (*p *= .003), 4 (*p *= .02) and 6 (*p *= .03) are located in roughly the same medial frontal regions. In this region, outflow was increased for the elderly in the 5-55 Hz (cluster 1), the 65-80 Hz (cluster 4), and the 80-97 Hz frequency bands. Clusters 2 (*p *= .003), 5 (*p *= .03), and 7 (*p *= .03) are located in the parietal region of the right hemisphere. Outflow was increased for the elderly in the 35-85 Hz (cluster 2), 15-35 Hz (cluster 5), and the 90-100 Hz (cluster 7) frequency bands. Cluster 3 is located in the dorsal aspects of the left frontal lobe and is significant for the 68-100 Hz frequency range. Cluster 8 is the only significant cluster with negative *t *values, i.e. the degree of outflow was stronger for younger compared to elderly individuals. This cluster covers parts of the right prefrontal lobe. Outflow was significantly reduced for the elderly people in the 1-12 Hz frequency range. While the results of the inflow pattern could be summarized into two main effects (see Figure 2 and 3), the results of the outflow pattern were numerous and less concise with at least eight significant main clusters and multiple additional smaller clusters. Therefore, we focused the following analysis on the inflow clusters to give a more detailed view on the inflow pattern.

### Correlation with psychometric data

In this step of analysis our goal was to investigate whether the two prominent clusters that showed significant age-related changes in inflow (the positive cluster in the medial temporal lobes and the negative cluster in the posterior part of the brain) correlate with behavioral performance. Therefore, we correlated the results of the psychometric testing with the degrees of the two significant clusters for each subject. We found several statistically significant correlations.

#### Positive cluster in the medial temporal lobe

Significant correlations were found for the Digit Symbol test (*p *< .001; *r *= -.45), Mosaic test (*p *= 0.004; *r *= -.39), the Benton test (*p *< .001; *r *= -.51), Figure Recall (*p *< .001, *r *= -.47) as well as the Trail Making test version A (*p *< .001; *r *= .45) and B (*p *= .003; *r *= .40) (For further details see table 1). In sum, stronger inflow in the medial temporal lobe was associated with weakened performance in executive function and cognitive speed (assessed with the Digit Symbol as well as the Trail Making Test A and B), visuoconstruction (Mosaic Test) as well as figural memory (Benton Test and Figure Recall).

**Table 1:** Correlation of the positive inflow clusters in the medial temporal lobes with cognitive performance (Pearson's product-moment correlation coefficient between the average degree of the respective cluster of all participants with their neuropsychological test score.

Neuropsychological Test	*R*	*P*
Digit Symbol Test	-.45	< .001*

Mosaic Test	-.39	.004*

Digit Span	-.16	.25

Benton Test	-.51	< .001*

Verbal Fluency	-.19	.17

Word List Learning	-.33	.02

Word List Delayed Recall	-.31	.03

Word List Recognition	-.23	.09

Figure Recall	-.47	< .001*

Trail Making Test A	.45	< .001*

Trail Making Test B	.4	.003*

To correct for multiple comparison, the threshold for a significant correlation was adjusted to the level of p = 0.0045 according to the Bonferroni-method)

Digit Symbol Test = Digit Symbol subtest of the German version of the Wechsler Adult Intelligence Scale; Mosaic test = Mosaic subtest of the German version of the Wechsler Adult Intelligence Scale; Digit Span = Digit Span subtest of the German version of the Wechsler Adult Intelligence Scale; Benton Test = correct answers of the Benton Visual Retention Test (revised form); Verbal Fluency = Sum score verbal fluency (semantic and phonemic) of the German version of the CERAD-NP-Plus test battery; Word List Learning = Subtest Word List-Learning of the German version of the CERAD-NP-Plus test battery; Word List Delayed Recall = Subtest Word List-Delayed Recall of the German version of the CERAD-NP-Plus test battery; Word List Recognition = Subtest Word List-Recognition of the German version of the CERAD-NP-Plus test battery; Figure Recall = Subtest Figure Recall of the German version of the CERAD-NP-Plus test battery; TMT-A = Trail Making Test - Version A; TMT-B = Trail Making Test - Version B

* p < .0045

#### Negative cluster in the posterior region

For this cluster, the correlations were significant for the Digit Symbol test (*p *< .001; *r *= .48), the Mosaic test (*p *< .001; *r *= .50), the Benton test (*p *= .003; *r *= .41), and the Trail Making test version B (*p *= .003; *r *= -.41) (For further details see table 2). In conclusion, decreased inflow in this region was associated with weakened performance in executive functions and cognitive speed (measured with the Digit Symbol Test and the Trail Making Test), visuoconstruction (Mosaic Test) as well as figural memory (Figure Recall).

**Table 2:** Correlation of the negative inflow cluster in posterior areas with cognitive performance (Pearson's product-moment correlation coefficient between the average degree of the respective cluster of all participants with their neuropsychological test score.

Neuropsychological Test	*R*	*P*
Digit Symbol Test	.48	< .001*

Mosaic Test	.5	< .001*

Digit Span	.09	.53

Benton Test	.4	.003*

Verbal Fluency	.09	.53

Word List Learning	.33	.02

Word List Delayed Recall	.3	.03

Word List Recognition	.27	.05

Figure Recall	.3	.03

Trail Making Test A	-.28	.05

Trail Making Test B	-.41	.002*

To correct for multiple comparisons, the threshold for a significant correlation was adjusted to the level of p = 0.0045 according to the Bonferroni method)

Digit Symbol Test = Digit Symbol subtest of the German version of the Wechsler Adult Intelligence Scale; Mosaic test = Mosaic subtest of the German version of the Wechsler Adult Intelligence Scale; Digit Span = Digit Span subtest of the German version of the Wechsler Adult Intelligence Scale; Benton Test = correct answers of the Benton Visual Retention Test (revised form); Verbal Fluency = Sum score verbal fluency (semantic and phonemic) of the German version of the CERAD-NP-Plus test battery; Word List Learning = Subtest Word List-Learning of the German version of the CERAD-NP-Plus test battery; Word List Delayed Recall = Subtest Word List-Delayed Recall of the German version of the CERAD-NP-Plus test battery; Word List Recognition = Subtest Word List-Recognition of the German version of the CERAD-NP-Plus test battery; Figure Recall = Subtest Figure Recall of the German version of the CERAD-NP-Plus test battery; TMT-A = Trail Making Test - Version A; TMT-B = Trail Making Test - Version B

* p < .0045

### Correlation between the significant inflow clusters

The correlation between the two clusters with age-related inflow changes (the positive cluster in the medial temporal lobe and the negative cluster in the precuneus/posterior cingulum region) was significant (r = -.43; p = .001).

## Discussion

The present study was conducted to answer two questions: 1) Is there neuromagnetic evidence for age-related alterations in long-range cortical networks and functional connectivity during the resting state and 2) do these potential changes correlate with the participants' cognitive performance? We will discuss the results of this study in relation to these questions.

Considering the first question, we identified brain regions with age-associated alterations in the functional connectivity with respect to the inflow and outflow characteristics of various brain regions. A strong inflow indicates that this area is driven by other regions whereas a strong outflow means that this area considerably influences the activity of other brain regions. Increasing age was associated with significantly more inflow in medial temporal areas and significantly less inflow in posterior parts of the brain. These alterations were found for the frequency bands from 8 to 100 Hz for the positive inflow cluster in the medial temporal lobe and for the frequency bands from 1 to 100 Hz in the negative inflow cluster in the posterior region. Regarding the outflow pattern, higher age was related to more outflow in medial frontal areas, in the parietal region of the right hemisphere as well as in dorsal areas of the left frontal lobe. Reduced outflow with increasing age was found in the right prefrontal lobe. These results, together with the scatterplots in Figure 2, strongly suggest distinct and progressive alterations in the functional organization of long-range cortical networks during healthy aging.

Interestingly, the areas with altered functional organization patterns largely overlap regions of the so-called "default-mode network", i.e., the regions with hemodynamic coupling during the resting state (e.g. [34,35]). This network is formed by a specific set of brain regions which is engaged when individuals are not focused on the external environment but is active when people are occupied with internally focused tasks like remembering or daydreaming. Buckner and colleagues [35] have argued that the default-mode network consists of several subsystems, namely the medial temporal lobe system, the medial frontal system as well as integrating systems like the posterior cingulate cortex/precuneus system. Alterations in the default network have already been reported for people with Alzheimer's disease [3,4] or corresponding genetic risk [36], mild cognitive impairment [5,37], and normal aging [3,17,38]. So far, evidence for alterations in the default network mainly comes from fMRI studies. However, the BOLD response lacks the temporal resolution needed to cover major bands of oscillatory brain activity. Hence, time-sensitive tools like MEG are suited to complement fMRI investigations and several studies have suggested that MEG recordings can be used to realiably analyze functional connectivity of the human brain (see e.g. [30,39-42]).

First, we found an age-related decrease in inflow in posterior parts of the brain, which would fit with the posterior cingulate cortex and the precuneus. This effect was found across the broad frequency range of 1-100 Hz and was not specific for a distinct frequency band. Thus, we suspect a rather general distortion of functional connectivity with this region. This is in line with results from Sperling and colleagues [17] who showed that cognitively intact elderly people with high amyloid burden exhibit disrupted default network activity, especially in the posterior cingulate cortex. The posterior cingulate cortex and the precuneus are prominent hubs in intrinsic functional connectivity [34] and they are vulnerable to early amyloid deposition [17]. It has been suggested that this region gathers information from the environment [34] and integrates input from different subsystems [35]. We observed a significant correlation of the inflow into this precuneus/posterior cingulate (PPC) region with increasing age. Further, this reduction was strongly associated with a decrease of cognitive performance in test of executive functions, cognitive speed, visuoconstruction and verbal memory. Working memory performance, however, did not correlate with the inflow into this region.

Second, we found a significant increase of inflow in the medial temporal region. In light of the default mode network, this region is part of the medial temporal lobe memory sub-system. It is active during internally directed cognition and is thought to be involved in declarative memory, especially in autobiographical memory [35,43,44]. Thus, an enhanced input into this region might go along with a stronger focus on autobiographical events and a reduced orientation to external stimulation. Here we found that inflow into this medial temporal region significantly correlates with the performance in cognitive speed, executive functions, visuoconstructive abilities and visual memory, but not with working memory.

Our results lead to the conclusion that there are several brain networks active during the resting state. Two resting state systems that are especially important for this study are the medial temporal lobe system and the posterior cingulum/precuneus system. The medial temporal lobe system is thought to reflect an internal orientation whereas the posterior cingulum/precuneus system seems to reflect a more external orientation [35]. More activity in one network is accompanied by reduced activity in the other [45,46]. During rest there are periodical and transient shifts in this network dominance, resulting in temporary shifts between internal and external foci of attention [45,46]. Accordingly, the strength of activation in these clusters and cognitive performance should be correlated in a way that the more external the focus, the better the performance on cognitive tests. This is exactly what we found: Higher inflow in the medial lobe subsystem (reflecting a stronger internal attention focus) was associated with weakened cognitive performance whereas stronger inflow in the posterior cluster (reflecting a stronger external attention focus) was related to better cognitive performance.

Furthermore, our results indicate that the balance between these two different subsystems underlies alterations with increasing age. Elderly people in general show more inflow in the medial temporal lobe subsystem and less inflow into the posterior cingulum/precuneus subsystem during the resting state, what we interpret as increased attention to internal processes and less attention to external stimulation.

It needs to be mentioned that the voxel grid that we used poses a limitation to this study. Due to the relatively large voxel size of 2 cm we cannot make assumptions on precisely localized brain regions since only the average activity of the 2 × 2 × 2 cm voxel was entered into the MVAR modeling. Therefore, it is also possible that we missed relevant brain activity which could have effected the modeling of the functional network.

## Conclusions

In summary, we found significant age-related alterations of functional resting-state connectivity. These are mainly characterized by reduced information input into the posterior cingulum/precuneus region together with an enhanced information flow to the medial temporal lobe. The pattern of these changes in functional cortical connectivity might indicate that the ongoing resting-state brain activity in elderly people is driven by attention to internal processes and autobiographical memories as opposed to attention to external stimulation. In our sample, these changes were associated with reduced performance in cognitive assessment batteries.

## Authors' contributions

WS carried out the data analysis and drafted the manuscript together with VL. VL conducted the study. ITK designed the study and participated in the discussion and interpretation of the results. NW participated in data analysis. TE participated in the discussion and interpretation of the results. ITK, NW and TE helped in drafting and revising the manuscript. All authors read and approved the final manuscript.
